# Promises to Pay

**Published:** 1900-11-03

**Authors:** 


### PROMISES TO PAY.

The necessity of certain forms of agreement being in writing was well illustrated in a case recently tried in the Darlington County Court in which a medical man claimed to recover fees for attendance on a patient who had died without estate, from a third party who had, as he understood, undertaken to pay him in case the patient should be unable so to do. The judge said he did not see how the plaintiff could recover the fees. In cases where medical men were called in to attend a patient and were to be paid by another person, they must have it in writing. The defendant might have said that it was all right, and that he did not mind being responsible, but it was necessary that it should be in writing. Commenting on this case, the *British Medical Journal* says that it is a wellestablished rule of law that a promise to answer for the debt or default of another, for which the other remains liable, must be in writing. If the defendant himself (either alone or jointly with others) had been primarily liable for the demand forming the subject of the promise—for example, if he had originally requested the attendance of the plaintiff on the patient and credit had been given him—then, although the services were rendered to another person, the verbal promise would probably have been sufficient.

